# Is there a Benefit of Multidisciplinary Cancer Team Meetings for Patients with Gastrointestinal Malignancies?

**DOI:** 10.1245/s10434-016-5178-3

**Published:** 2016-03-22

**Authors:** Yara L. Basta, Onno L. Baur, Susan van Dieren, Jean H. G. Klinkenbijl, Paul Fockens, Kristien M. A. J. Tytgat

**Affiliations:** Department of Gastroenterology and Hepatology, Academic Medical Center, Amsterdam, The Netherlands; Gastrointestinal Oncology Center (GIOCA), Academic Medical Center, Amsterdam, The Netherlands; Department of Surgery, Academic Medical Center, Amsterdam, The Netherlands; Department of Clinical Epidemiology, Academic Medical Center, Amsterdam, The Netherlands; Department of Surgery, Gelre Ziekenhuizen, Apeldoorn, The Netherlands

## Abstract

**Background:**

Multidisciplinary cancer team meetings are intended to optimize the diagnosis of a patient with a malignancy. The aim of this study was to assess the number of correct diagnoses formulated by the multidisciplinary team (MDT) and whether MDT decisions were implemented.

**Methods:**

In a prospective study, data of consecutive patients discussed at gastrointestinal oncology MDT meetings were studied, and MDT diagnoses were validated with pathology or follow-up. Factors of influence on the correct diagnosis were identified by use of a Poisson regression model. Electronic patient records were used to assess whether MDT decisions were implemented, and reasons to deviate from these decisions were hand-searched within these records.

**Results:**

In 74 MDT meetings, 551 patients were discussed a total of 691 times. The MDTs formulated a correct diagnosis for 515/551 patients (93.4 %), and for 120/551 (21.8 %) patients the MDT changed the referral diagnosis. Of the MDT diagnoses, 451/515 (87.6 %) were validated with pathology. Patients presented to the MDT by their treating physician were 20 % more likely to receive a correct diagnosis [relative risk (RR) 1.2, 95 % confidence interval (CI) 1.1–1.5], while the number of patients discussed or the duration of the meeting had no influence on this (RR 1.0, 95 % CI 0.99–1.0; RR 1.0, 95 % CI 0.9–1.1; resp.). MDT decisions were implemented in 94.4 % of cases. Deviations of MDT decisions occurred when a patient’s wishes or physical condition were not taken into account.

**Conclusions:**

MDTs rectify 20 % of the referral diagnoses. The presence of the treating physician is the most important factor to ensure a correct diagnosis and adherence to the treatment plan.

Multidisciplinary cancer team meetings are intended to optimize the diagnosis for patients with malignancies, thereby increasing the likelihood that patients will receive the best possible care.^1^ A multidisciplinary team (MDT) consists of healthcare professionals from different disciplines who offer their specific service and contribute to the best care for each individual patient.^2^–^4^ MDTs have been implemented worldwide to increase the quality of care; however, the extent to which gastrointestinal oncology MDTs actually improve the quality of care remains undetermined.

In many other countries, including The Netherlands, MDTs have become mandatory,^3^,^5^,^6^ which has made the evaluation of the influence of MDTs on the quality of care increasingly difficult. There is no comparable control group for patients evaluated by an MDT, rendering randomized controlled trials impossible. To assess the quality of MDTs, different studies have suggested evaluating survival; however, these studies are likely subject to bias. When a prospective intervention group is compared with an historical cohort, differences in treatment over time may influence results.^1^,^6^–^8^ Since the use of a prospective control group is infeasible, it is difficult to determine the influence of MDTs on the quality of care with a direct performance measure (e.g. survival). Instead, Kurpad et al. and Lamb et al. suggested an indirect measure may be used to evaluate the impact of MDTs—the decision-making process,^1^,^4^,^9^ which can be assessed using the number of correct diagnoses formulated by the MDT.^1^,^4^,^9^–^13^

To date, studies describing MDTs or their decision-making process do not relate to gastrointestinal malignancies, are of retrospective design, or involve a subjective evaluation by medical professionals.^3^,^9^,^10^,^14^,^15^ However, some variables described in these studies can be used in a prospective study, i.e. the presence of a chairperson, the presence of the treating physician, the number of patients discussed, time pressure, interruptions such as pagers frequently ringing, and the presence of all necessary medical specialties.^2^,^4^,^16^

To ensure that patients receive the best possible care, it is not only important to assess whether the correct diagnosis is made but also to determine whether the MDT decisions are implemented; approximately 18 % of MDT decisions are not implemented.^11^ Understanding the reasons for not implementing MDT decisions could improve the quality of care and should also be taken into account when studying the decision-making process of an MDT.^3^,^4^,^11^,^17^

The primary objective of this study was to evaluate the decision-making process of a gastrointestinal cancer MDT at a tertiary referral center, together with factors influencing this process. Since time pressure is considered to be very influential on the decision-making process, factors influencing the duration of the MDTs and individual patient discussions were also evaluated.^2^,^4^,^16^ The secondary aim of this study was to evaluate whether MDT decisions were implemented and which factors were responsible for not implementing these decisions.

## Methods

### Setting

This prospective study was conducted at a fast-track clinic (FTC) for patients with (suspected) gastrointestinal malignancies. The FTC is a tertiary referral center located in a university hospital in The Netherlands. Four tumor-specific MDT meetings are held from Monday to Thursday: hepatocellular carcinoma (HCC), colorectal carcinoma (CRC), esophageal and gastric cancer (ESOGAS) and pancreatobiliary tumors (PB). Each meeting is attended by a tumor-specific MDT consisting of specialized gastrointestinal cancer nurses and representatives from each involved specialty (specialty physicians): surgery, gastroenterology, medical oncology, radiation oncology, radiology, pathology, and nuclear medicine. After referral by a medical specialist, imaging is re-reviewed by a specialized radiologist. If the imaging is missing or not current enough, additional imaging is performed. The additional imaging is performed before the MDT meeting, on the day of the appointment. Pathology samples are requested after each referral and are preferably assessed before the meeting; however, since the waiting time to the FTC is 6 days or less, pathology can be delayed.

New patients spend one full day at the FTC. In the morning they are seen by the treating physician (either a surgeon or gastroenterologist) who evaluates their symptoms and performance status. At noon, the tumor-specific MDT convenes for a lunch meeting, and patients are (preferably) presented by the treating physician. During this meeting, the MDT either confirms or rectifies the referral diagnosis and formulates a treatment plan for each individual patient. All decisions made by the MDT are documented ‘real-time’ in a shared electronic patient record (EMR). The EMR is accessible to all specialty physicians and the specialized nurses, enabling them to consult the notes documented during the MDT meeting and ensuring all convey the same information to the patient.

In the afternoon following the meeting, the treating physician discusses the MDT diagnosis and treatment plan with the patient. If a diagnosis or treatment plan cannot be formulated during the MDT meeting, the patient is discussed again during a subsequent MDT meeting. For the purpose of this study, these patients are defined as follow-up patients. Generally, follow-up patients do not spend the entire day at the FTC.

### Data Collection

The decision-making process of the MDT was investigated by assessing the number of correct diagnoses formulated by the MDT, as well as the number of rectified referral diagnoses. An independent researcher records the MDT diagnosis during the meeting and compares this with the referral diagnosis. Diagnoses formulated by the MDT were validated either by pathology (preferred) or imaging and laboratory results. Patients with a diagnosis not confirmed by pathology, or with a benign diagnosis, were observed during follow-up. The variables recorded during the meetings are documented in Table 1. Clinical data gathered from the EMR included age, sex, MDT diagnosis and stage, referral diagnosis, and pathology results.

**Table 1:** Variables recorded during meeting

Variable	Poisson analysis (correct diagnosis)	Linear regression (duration of meeting)	Linear regression (duration of patient discussion)
Presence/absence of the specialty physicians^a^	–	+	+
Presence of the treating physician	+	–	+
Total number of people present	–	+	–
Presence of a chairperson	+	+	+
Number of interruptions during the meeting^b^	+	–	+
Duration of the meeting	+	+^c^	+^c^
Duration of individual patient discussion (min)	+	–	–
Follow-up patient, yes or no	–	–	+
Total number of patients discussed	+	+	–
Need for additional imaging	+	–	–
Change of referral diagnosis or treatment	–	–	+

^a^Including not only specialty physicians but also researchers, nurses, students, and residents

^b^Interruptions consisted of doctors arriving late or leaving early, and pagers ringing. ‘Late arrival’ was defined as arrival after the start of the meeting or delaying the scheduled start of the meeting by more than 2 min

^c^Delay of the start of the meeting, in minutes

The data of patients discussed at consecutive MDT meetings were collected at two time intervals, with the first interval being from December 2012 to March 2013. From March to April 2013 a software upgrade was performed, comprising a new form in the EMR to facilitate documenting the decisions made by the MDT. Three months later, the form was adapted to increase user friendliness. Although the same information was documented in both forms, the new form differed in structure from the previous form, and more input fields needed to be filled in, e.g. additional input fields for the treating physician and the chairperson, as opposed to one general input field. Since this could potentially introduce a bias of the time measurements of the patient discussions, no data were included during this period.^18^ From September to December 2013, data collection for the second interval took place. Patient characteristics of the first and second periods were compared using a Chi square test to evaluate the presence of any differences and to ensure the two groups could be analyzed as a single cohort.

Adherence to MDT decisions was assessed during follow-up using all available information, e.g. charts, medical letters. If treatment differed from the decision of the MDT, hospital records where examined to determine why these changes in the treatment plan were made.

### Data Analysis

Statistical analysis was performed using SPSS version 21.0 software (IBM Corporation, Armonk, NY, USA). To approximate the relative risk (RR) of a correct diagnosis at the first MDT meeting, a multivariable modified Poisson regression analysis was used. Included variables were based on clinical relevance and were supported by literature (Table 1).^2^,^4^,^16^ With the Poisson analysis, the error for the approximated RR can be overestimated; therefore, a robust error variance procedure known as *sandwich estimation* was used to obtain confidence intervals (CI).^19^ The model was corrected for the different tumor-specific MDTs.

To identify factors influencing the duration of the MDTs and the discussion of the individual patient, two multivariate linear regression models were used. Included variables were based on clinical relevance, supported by literature (Table 1).^2^,^4^,^16^ Both models were corrected for the differences between the various tumor-specific MDTs.

### Ethics Committee Approval

Due to the observational nature of this study, the local Medical Ethics Committee determined that formal approval was not required.

## Results

Seventy-four gastrointestinal cancer MDT meetings took place in 6 months, with a mean duration of 63 min (SD ± 14). In total, 691 discussions took place for 551 new patients, of which 140/551 patients were discussed in two or more MDT meetings. The mean discussion time per new patient was 05:34 min (SD ± 2; 95 % CI 5:18–5:49), and 4:20 min (SD 2.52; 95 % CI 4:00–4:41) per follow-up patient. Patient characteristics were similar during both time periods, with the exception of the number of HCC patients discussed (Table 2).

**Table 2:** Patient characteristics

Characteristics	First period^a^	Second period^b^	*p* value
No. of patients^c^	277	274	
Age [years; mean (SD)]	63 (11.6)	66 (24.8)	0.24
Male [*n* (%)]	178 (64)	173 (63)	0.49
Second opinion	39 (14)	64 (23)	0.87
Diagnosis at first MDTM [*n* (%)]			0.39
Correct diagnosis	238 (86)	240 (88)	
Incorrect diagnosis	8 (3)	10 (4)	
Treatment intent [*n* (%)]			0.26
Curative	105 (38)	123 (45)	
Palliative	78 (28)	78 (29)	
Unknown	72 (26)	56 (20)	
Not applicable	22 (8)	17 (6)	
Tumor-specific MDT [*n* (%)]			
HCC	31 (11)	19 (7)	0.046
CRC	56 (20)	56 (20)	0.95
ESOGAS	74 (27)	87 (32)	0.19
PB	116 (42)	112 (41)	0.81
Time/MDTM [hours; mean (SD)]			
HCC	45:54 (16)	39:16 (7)	0.007
CRC	50:32 (12)	53:44 (17)	0.43
ESOGAS	63:00 (11)	62:00 (11)	0.13
PB	71:00 (7)	73:00 (9)	0.18
Time/patient [min; mean (SD)]			
HCC	06:28 (2)	07:23 (3)	0.34
CRC	05:34 (2)	06:09 (3)	0.30
ESOGAS	05:32 (2)	05:09 (3)	0.40
PB	04:27 (1)	04:59 (2)	0.056

*p* values were calculated using a Chi square test

^a^First period: 27 December 2012 to 12 March 2013

^b^Second period: 24 September 2013 to 5 December 2013

^c^Only individual patients were considered in this table

*HCC* hepatocellular carcinoma, *CRC* colorectal carcinoma, *ESOGAS* esophageal and gastric cancer, *PB* pancreatic and biliary tumors, *SD* standard deviation, *MDT* multidisciplinary team, *MDTM* multidisciplinary team meeting

**Fig. 1:**
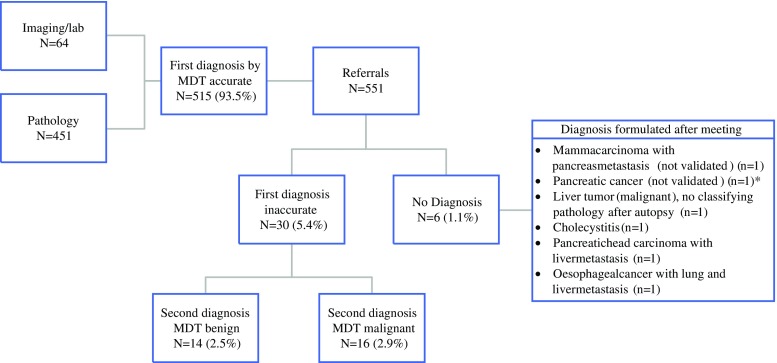
Diagnoses formulated by the MDT. In total, the MDT formulated a diagnosis for 545 patients—515 (93.5 %) accurate diagnoses, of which 451 were validated with pathology, and 30 inaccurate diagnoses. The MDT formulated a new diagnosis for these patients after review of additional information. Eventually, 14 patients who had previously received a malignant diagnosis turned out to have benign disease. *Diagnosis uncertain and treatment never initiated. *MDT* multidisciplinary team

The MDTs formulated a diagnosis for 545/551 patients (Fig. 1). In 515/551 (93.5 %) patients, the MDT diagnosis was correct, and 449/551 (81.5 %) of the diagnoses were formulated at the first MDT meeting. In total, 87.8 % (*n* = 451/545) of the diagnoses were validated with pathology. In 64/545 patients (11.7 %) the diagnosis was based solely on imaging and laboratory results, none of which were changed during follow-up (median 9.0 months; minimum–maximum: 0–35). For the 6/551 (1.1 %) patients in whom the MDT could not formulate a diagnosis, additional biopsies and imaging were performed; however, still no consensus could be reached and no treatment plan was proposed. Of the 30/551 patients incorrectly diagnosed by the MDT, 14/551 (2.5 %) had a benign diagnosis (cholecystitis, *n* = 4; pancreatitis, *n* = 3; gallstones, *n* = 1; diverticulitis, *n* = 1; lipoma, *n* = 1; leiomyoma, *n* = 1; hemangioma, *n* = 1; polyp, *n* = 2). For the remaining 16/551 patients, a malignant diagnosis was formulated following pathological investigation (*n* = 7), surgery (*n* = 7), or additional imaging (*n* = 2).

**Fig. 2:**
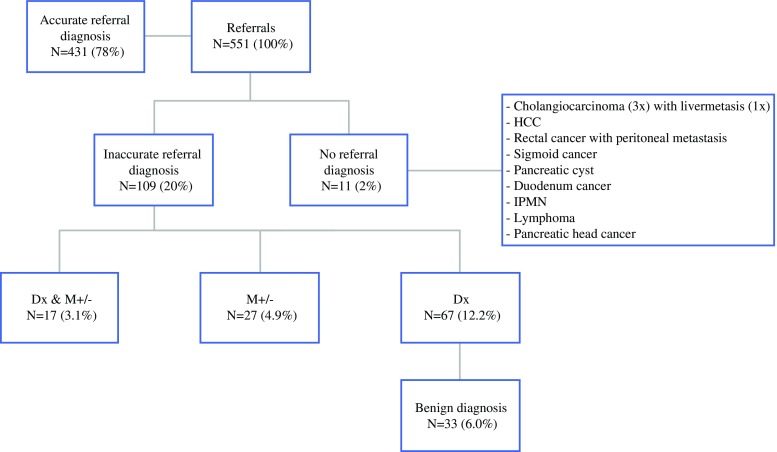
Changes in referral diagnosis. Of the 551 patients referred, the MDT diagnosis was the same as the referral diagnosis in 431 patients. Eleven patients were referred without a diagnosis, and the MDT diagnosed all these patients; three were diagnosed with a cholangiocarcinoma, of which one patient also had liver metastasis, two patients had a benign diagnosis, and the remaining six patients had various malignancies. Patients referred without a diagnosis were suspected of having a malignancy. For 67 patients the diagnosis alone was changed; of these patients, 33 had a benign diagnosis. *Dx* diagnosis, *M*± change in staging of disease, *MDT* multidisciplinary team

The MDT changed the referral diagnosis for 121/551 (22.0 %) patients. In one patient, the referral diagnosis was incorrectly changed from esophageal to gastric cancer. Of the 120/551 (21.8 %) patients with a rectified diagnosis, 11 patients were referred without a diagnosis, but with high suspicion of malignancy. For two of these patients, the MDT formulated a benign diagnosis. For the 109/120 patients referred with a diagnosis, both diagnosis and stage were rectified for 17/551 (3.1 %) patients. Of these patients, 14 initially diagnosed with localized disease were reclassified to metastatic disease, and four patients referred with metastatic disease were reclassified to localized disease. Stage alone was changed in 27/551 patients (4.9 %), of which five patients referred with metastatic disease were rediagnosed to have localized disease. Fifteen patients initially diagnosed with localized disease were rediagnosed to metastatic disease. The remaining seven patients had more extensive metastatic disease than as diagnosed by the referring physician. Diagnosis alone was rectified in 67/551 (12.2 %) patients. Of these patients, 33/551 (6.0 %) were rediagnosed to benign disease (Fig. 2). Rectified diagnoses were more often observed for patients discussed by the PB MDT (32.9 %) compared with the other MDTs.

Following correction for the different MDT meetings, the treating physician was the most influential factor to ensure a correct diagnosis: patients were 20 % more likely to receive a correct diagnosis (RR 1.2, 95 % CI 1.01–1.47). Patients were less likely to be diagnosed correctly when additional tests were needed after the MDT meeting (RR 0.8, 95 % CI 0.76–0.93), and the number of patients or the duration of the meeting did not influence this (95 % CI 0.98–1.0) [Table 3]. The duration of a patient’s discussion increased when the MDT changed the referral diagnosis (0:31 min/patient, 95 % CI 0:02–1:01), or when a chairperson was present (1:14 min/patient, 95 % CI 0:32–1:24) [Table 4]. However, these variables did not influence the probability of a correct diagnosis (95 % CI 0.98–1.01) [Table 3].

**Table 3:** Poisson analyses of variables influencing correct diagnosis

Variable	RR	95 % CI	*p* value
Treating physician (yes)	1.2	1.02–1.47	0.046
Duration of patient discussion	1.0	0.98–1.00	0.24
Duration of MDTM	1.0	0.99–1.00	0.26
Additional tests needed (yes)	0.8	0.76–0.93	<0.001
Presence of chairperson (yes)	1.0	0.97–0.10	0.31
Total number of patients	1.0	0.98–1.01	0.29
Interruptions	1.0	0.99–1.01	0.67

Multivariable analysis with modified Poisson regression to estimate the RR

The variable ‘Treating physician (yes)’ indicates that the treating physician has presented the patient to the MDT; the variable ‘Additional tests needed (yes)’ indicates the need for additional tests; and the variable ‘Presence of chairperson (yes)’ indicates a chairperson was present

*RR* relative risk, *CI* confidence interval, *MDT* multidisciplinary team, *MDTM* multidisciplinary team meeting

Corrected for tumor-specific MDTs

**Table 4:** Linear regression model of variables influencing duration

Variable	Influence on duration (min)	95 % CI	*p* value
*Duration of MDTM*			
Patients	2:20	1:29 to 3:10	<0.001
Interruptions^a^	0:14	0:03 to 0:26	0.015
Minutes late starting meeting	−0:53	−2:10 to 0:22	0.16
Absence of medical specialist	3:24	−1:48 to 8:37	0.19
Presence of chairperson	0:57	−4:19 to 6:14	0.71
Total physicians present	0:54	0:02 to 1:46	0.042
Total non-physicians present	−0:07	−1:46 to 0:56	0.79
Tumor type			
HCC	Reference group		
CRC	1:51	−4:32 to 8:15	0.56
ESOGAS	0:52	−5:30 to 7:14	0.78
PB	4:34	−4:49 to 3:58	0.33
*Time per patient* ^b^			
Follow-up patient	−1:47	−2:10 to −1:18	<0.001
Interruptions	−0:03	−0:07 to 0:10	0.16
Minutes late starting meeting	0:05	−0:01 to 0:12	0.090
Presence of chairperson	0:35	0:08 to 1:03	0.010
Treating physician	−0:07	−0:58 to 0:44	0.59
Absence of medical specialist	−0:03	−0:30 to 0:21	0.81
Changing diagnosis	0:28	0:02 to 1:01	0.056
Changing treatment	1:14	0:32 to 1:24	0.001
Tumor type			
HCC	Reference group		
CRC	−1:31	−2:18 to −0:38	0.001
ESOGAS	−1:36	−2:21 to −0:46	<0.001
PB	−2:38	−3:21 to −1:51	<0.001

Linear regression model. The variable ‘Treating physician’ indicates whether the treating physician has presented the patient to the MDT (yes or no)

^a^Interruptions per minute, corrected for total duration of MDTM

^b^All patients included

*CI* confidence interval, *MDT* multidisciplinary team, *MDTM* multidisciplinary team meeting, *HCC* hepatocellular carcinoma, *CRC* colorectal carcinoma, *ESOGAS* esophageal and gastric cancer, *PB* pancreatic and biliary tumors

A treatment plan was formulated for 542 patients. Fifteen patients were excluded from analyses because it was unclear what treatment they received (*n* = 6) or no treatment plan was formulated (*n* = 9). Of the remaining patients, 31/536 (5.8 %) received a different treatment than advised by the MDT. Deviation occurred when patients’ wishes or physical condition were not taken into account: 15 patients preferred a different treatment or no treatment, and 14 patients were physically unable to undergo the preferred treatment, of which four patients died before treatment could be initiated and four patients had progressed further than was assumed by the MDT. Treatment for one patient changed after a second opinion from another specialized oncology center, and two patients were incorrectly diagnosed by the MDT. After their diagnosis was corrected, these patients were treated accordingly.

## Discussion

This is the first study that has prospectively evaluated how often MDTs formulate a correct diagnosis and the factors that influence this. The value of a correct diagnosis lies in the assumption that a correct diagnosis will lead to a proper treatment plan, avoiding over- or under-treatment.^4^^,^^20^ The present study shows that MDTs formulate a correct diagnosis and stage for 94 % of the referred patients, and the MDT rectified the referral diagnosis and stage in 22 % of the evaluated patients. The presence of the treating physicians was the most influential variable to ensure a correct diagnosis.

MDTs are increasingly initiated in order to, ultimately, improve patient outcomes.^1^,^4^,^15^,^21^,^22^ The development of multimodal treatments further emphasizes the need for a multidisciplinary approach. Although it may seem unquestionable that MDTs have a positive effect on the patient outcomes, this remains difficult to evaluate.^4^,^9^,^15^ This difficulty is inherent to comparative studies of healthcare outcomes and the fact MDTs have become mandatory in many Western countries, limiting the use of a prospective control group.^3^,^5^,^6^,^15^ Three studies from the Johns Hopkins 1-day diagnostic clinic in the US tried to evaluate the effect of MDTs by determining how often referral diagnoses and treatment plans are altered after evaluation by a specialized MDT.^15^,^22^,^23^ The study by Pawlik et al. had a prospective design and found that a pancreas cancer MDT altered the initial diagnosis in 22.2 % of evaluated cases.^23^ The retrospective design study by Zhang et al. observed that the MDT of a specialized liver clinic altered the diagnosis in 18.4 % of evaluated cases,^22^ while the study by Sundi et al. also had a retrospective design. These investigators found that 28.7 % of men referred with prostate cancer had a change in their risk category or stage.^15^ Most of the altered diagnoses described in these studies were formulated after re-review of imaging and pathology, or new findings resulting from additional diagnostic tests. Although these studies report the changes in referral diagnoses, they do not explicitly report if the diagnosis was correctly changed, nor do they report variables of influence on formulating an accurate diagnosis.^15^,^22^,^23^

Some literature on the variables of influence on decision making of MDTs is available; however, these factors have not been previously studied for gastrointestinal cancer MDTs. In a review of the literature, Lamb et al. noticed that the treating physician contributes to an improved decision-making process,^12^ and the present study confirmed this. The presence of a treating physician with pre-existing knowledge of the patient increases the probability of a correct diagnosis (RR 1.2). This physician is also most likely to know the patients’ wishes and ensures an individualized treatment plan, emphasizing the importance of his presence. In a single case study design studying a large gynecology cancer MDT, Lanceley et al. found that time pressure, absence of medical specialists, and lack of leadership negatively influenced the decision-making process.^13^ Unexpectedly, in this study the duration of the MDT meeting and the number of patients discussed (perceived time pressure) did not seem to influence the probability of a correct diagnosis. Furthermore, our results did not show that the presence of a chairperson or the absence of a medical specialist influenced the probability of a correct diagnosis; however, we feel it is important to stress that an MDT will likely function better when all involved specialty physicians required to diagnose the patient are present.^11^,^17^

### Limitations

The number of variables included in a linear or logistic regression model is restricted to the number of events divided by 10. The MDT did not formulate a (correct) diagnosis at the first meeting for 66 patients, restricting the number of variables to six or seven. Factors of influence of the decision-making process could have been missed by this restriction. Of the evaluated patients, 22 % received a different diagnosis by the MDT; however, due to the selected patient group, it is unclear how these results can be extrapolated to other MDTs for either different malignancies or benign diseases. It is possible the effect of MDTs is greatest for patients with complex disease, such as gastrointestinal malignancies. Furthermore, for many of the referrals it was unclear whether the referral diagnosis was formulated by an individual physician or a (less specialized) MDT. This can potentially create a bias in our results.

## Conclusions

The evidence proving the added value of MDTs in cancer care is growing and the results of this study further endorse this hypothesis. To improve the quality of MDTs, adequate documentation of decisions made by the MDT accessible by all involved physicians and nurses is needed. This not only improves communication between different specialties but also ensures all physicians convey the same MDT advice to the patient. In our opinion, a shared EMR is necessary for a well-functioning MDT, and both current and future EMRs should incorporate this. However, the most influential variable to ensure an accurate diagnosis and to take into account both patient preferences and performance state is relatively simple and can easily be extrapolated to MDTs in other countries. Every patient discussed by an MDT should be presented by a physician who has seen and talked to the patient.

## References

[1] Lamb BW, Sevdalis N, Taylor C, Vincent C, Green JS (2012). Multidisciplinary team working across different tumour types: analysis of a national survey. Ann Oncol..

[2] Lamb BW, Sevdalis N, Mostafid H, Vincent C, Green JS (2011). Quality improvement in multidisciplinary cancer teams: an investigation of teamwork and clinical decision-making and cross-validation of assessments. Ann Surg Oncol..

[3] Lamb BW, Brown KF, Nagpal K, Vincent C, Green JS, Sevdalis N (2011). Quality of care management decisions by multidisciplinary cancer teams: a systematic review. Ann Surg Oncol..

[4] Kurpad R, Kim W, Rathmell WK (2011). A multidisciplinary approach to the management of urologic malignancies: does it influence diagnostic and treatment decisions?. Urol Oncol..

[5] Signaleringscommissie Kanker van KWF Kankerbestrijding. Kanker in Nederland, Trends, prognoses en implicaties voor zorgvraag [Signalling Comittee Cancer of the Dutch Cancer Society. Cancer in the Netherlands: Trends, prognosis and implications for the demand for care]. Amsterdam: Report of the Dutch Cancer Society; 2004.

[6] Department of Health (2004). Manual for cancer services 2004.

[7] Kesson EM, Allardice GM, George WD, Burns HJ, Morrison DS (2012). Effects of multidisciplinary team working on breast cancer survival: retrospective, comparative, interventional cohort study of 13 722 women. BMJ..

[8] Stephens MR, Lewis WG, Brewster AE (2006). Multidisciplinary team management is associated with improved outcomes after surgery for esophageal cancer. Dis Esophagus..

[9] Lamb BW, Green JS, Benn J, Brown KF, Vincent CA, Sevdalis N (2013). Improving decision making in multidisciplinary tumor boards: prospective longitudinal evaluation of a multicomponent intervention for 1,421 patients. J Am Coll Surg..

[10] Hong NJL, Gagliardi AR, Bronskill SE, Paszat LF, Wright FC (2010). Multidisciplinary cancer conferences: exploring obstacles and facilitators to their implementation. J Oncol Pract..

[11] Blazeby JM, Wilson L, Metcalfe C, Nicklin J, English R, Donovan JL (2006). Analysis of clinical decision-making in multi-disciplinary cancer teams. Ann Oncol..

[12] Lamb B, Green JS, Vincent C, Sevdalis N (2011). Decision making in surgical oncology. Surg Oncol..

[13] Lanceley A, Savage J, Menon U, Jacobs I (2008). Influences on multidisciplinary team decision-making. Int J Gynecol Cancer..

[14] Newman EA, Guest AB, Helvie MA (2006). Changes in surgical management resulting from case review at a breast cancer multidisciplinary tumor board. Cancer..

[15] Sundi D, Cohen JE, Cole AP (2015). Establishment of a new prostate cancer multidisciplinary clinic: format and initial experience. Prostate..

[16] Fleissig A, Jenkins V, Catt S, Fallowfield L (2006). Multidisciplinary teams in cancer care: are they effective in the UK?. Lancet Oncol..

[17] Jalil R, Ahmed M, Green JS, Sevdalis N (2013). Factors that can make an impact on decision-making and decision implementation in cancer multidisciplinary teams: an interview study of the provider perspective. Int J Surg..

[18] Ngwenyama O, Guergachi A, McLaren T (2007). Using the learning curve to maximize IT productivity: A decision analysis model for timing software upgrades. Int J Prod Econ..

[19] Zou G (2004). A modified poisson regression approach to prospective studies with binary data. Am J Epidemiol..

[20] Davies AR, Deans DA, Penman I (2006). The multidisciplinary team meeting improves staging accuracy and treatment selection for gastro-esophageal cancer. Dis Esophagus..

[21] Taylor C, Munro AJ, Glynne-Jones R (2010). Multidisciplinary team working in cancer: what is the evidence?. BMJ..

[22] Zhang J, Mavros MN, Cosgrove D (2013). Impact of a single-day multidisciplinary clinic on the management of patients with liver tumours. Curr Oncol..

[23] Pawlik TM, Laheru D, Hruban RH (2008). Evaluating the impact of a single-day multidisciplinary clinic on the management of pancreatic cancer. Ann Surg Oncol..

